# Elimination of Alcohol by the Respiration

**Published:** 1875-10

**Authors:** 


ELIMINATION OF ALCOHOL BY THE RESPIRATION.—Aug. Schmidt (*Centralblatt,* 1875, p. 371) states that, having read Henbach's late observations showing that little or no alcohol appeared in the urine of febrile patients to whom it had been freely administered, he resolved to submit the expired air to renewed investigation, and the result of his experiments is, that when 50 ccm. of absolute alcohol have been consumed, at most only a trace can be discovered in the expired air. This is in full accordance with the statements of Anstie and Dupre.—*London Lancet.*

